# Chromosome landmarks and autosome-sex chromosome translocations in *Rumex hastatulus*, a plant with XX/XY1Y2 sex chromosome system

**DOI:** 10.1007/s10577-014-9446-4

**Published:** 2014-11-14

**Authors:** Aleksandra Grabowska-Joachimiak, Adam Kula, Tomasz Książczyk, Joanna Chojnicka, Elwira Sliwinska, Andrzej J. Joachimiak

**Affiliations:** 1 Department of Plant Breeding and Seed Science, University of Agriculture in Cracow, Łobzowska 24, 31-140 Cracow, Poland; 2 Department of Environmental Stress Biology, Institute of Plant Genetics of the Polish Academy of Sciences, Strzeszyńska 34, 60-479 Poznań, Poland; 3 Department of Plant Genetics, Physiology and Biotechnology, University of Technology and Life Sciences, Kaliskiego Ave. 7, 85-789 Bydgoszcz, Poland; 4 Department of Plant Cytology and Embryology, Institute of Botany, Jagiellonian University, Gronostajowa 9, 30-387 Cracow, Poland

**Keywords:** *Rumex hastatulus*, Sex chromosomes, Karyotype, FISH, rDNA, C-banding/DAPI

## Abstract

*Rumex hastatulus* is the North American endemic dioecious plant with heteromorphic sex chromosomes. It is differentiated into two chromosomal races: Texas (T) race characterised by a simple XX/XY sex chromosome system and North Carolina (NC) race with a polymorphic XX/XY1Y2 sex chromosome system. The gross karyotype morphology in NC race resembles the derived type, but chromosomal changes that occurred during its evolution are poorly understood. Our C-banding/DAPI and fluorescence in situ hybridization (FISH) experiments demonstrated that Y chromosomes of both races are enriched in DAPI-positive sequences and that the emergence of polymorphic sex chromosome system was accompanied by the break of ancestral Y chromosome and switch in the localization of 5S rDNA, from autosomes to sex chromosomes (X and Y2). Two contrasting domains were detected within North Carolina Y chromosomes: the older, highly heterochromatinised, inherited from the original Y chromosome and the younger, euchromatic, representing translocated autosomal material. The flow-cytometric DNA estimation showed ∼3.5 % genome downsizing in the North Carolina race. Our results are in contradiction to earlier reports on the lack of heterochromatin within Y chromosomes of this species and enable unambiguous identification of autosomes involved in the autosome-heterosome translocation, providing useful chromosome landmarks for further studies on the karyotype and sex chromosome differentiation in this species.

**Electronic supplementary material:**

The online version of this article (doi:10.1007/s10577-014-9446-4) contains supplementary material, which is available to authorized users.

## Introduction

Heteromorphic sex chromosomes are known only from a limited number of dioecious plants. The scattered phylogenetic distribution of taxa with these chromosomes suggested that chromosomal sex determination evolved in these organisms independently (Ming et al. 2011). Angiosperms are of particular interest for empirical studies of sex chromosome evolution, because they evolved separate sexes repeatedly and relatively recently (Charlesworth 2002). Moreover, they developed, like animals, either simple (XX/XY) or complex (polymorphic) sex chromosome systems (Matsunaga and Kawano 2001, Vyskot and Hobza 2004, Jamilena et al. 2008, Weingartner and Delph 2014). Two plant genera, *Humulus* and *Rumex*, revealed the presence of two different sex chromosome systems—XX/XY and XX/XY1Y2 (Parker and Clark 1991, Mariotti et al. 2009). Most probably, polymorphic sex chromosomes have evolved in these taxa from XX/XY ones by chromosome translocations (Grabowska-Joachimiak et al. 2011, Navajas-Pérez 2012). In comparison to XX/XY relatives, the lower chromosome counts in XX/XY1Y2 species may support this hypothesis.

The large genus *Rumex* of the Polygonaceae family contains a number of dioecious species showing sex chromosomes and two different sex determination systems (active-Y and X/autosome balance). In addition, monoecious, gynodioecious, hermaphroditic and polygamous species are also observed. For these reasons, the genus serves as an excellent model for studies on evolution of reproductive systems, sex-determining mechanisms and sex chromosomes in plants. The phylogenetic analyses demonstrated that species with heteromorphic sex chromosomes are clustered in two *Rumex* subgenera, *Acetosa* and *Acetosella*, and that the majority of dioecious species are represented by *R. acetosa* and its close relatives (Navajas-Pérez et al. 2005a). Two different sex chromosome systems were observed only within the subgen. *Acetosa*, in which multiple sex chromosomes appeared independently in European (*R. acetosa* and its relatives) and American (*R. hastatulus*) lineages (Navajas-Pérez et al. 2009a). All representatives of the European XX/XY1Y2 lineage analysed so far showed heteropicnotic Y chromosomes, enriched in DAPI-positive heterochromatin (Navajas-Pérez 2012). According to Quesada del Bosque et al. (2011), male sex chromosomes of *R. hastatulus* XX/XY1Y2 race are euchromatic and deprived of DAPI segments.

Since the time when Shibata et al. (1999) discovered RAYSI, the first satellite sequence massively amplified within heterochromatin portions of *R. acetosa* Y chromosomes, several other Y-associated repetitive sequences have been described, mainly in European XX/XY1Y2 *Rumex* lineage. Some of them were homologous to the RAYSI and located exclusively within Y chromosomes (RAYSII and RAYSIII), and another (RAE180) within Y chromosomes and autosomes (Shibata et al. 2000, Mariotti et al. 2009, Grabowska-Joachimiak et al. 2012, Navajas-Pérez 2012). From those satellite DNA sequences, only RAE180 proved to be poorly represented in the karyotype of *R. hastatulus*, but not within Y chromosomes. Due to different variants of this sequence found on the autosomes of all XX/XY *Rumex* species analysed so far, it was suggested that RAE180 marked the origin of dioecy in this genus (Navajas-Pérez et al. 2009b). Most probably the common ancestor of dioecious species possessed a set of divergent, autosomally located variants of this sequence (Quesada del Bosque et al. 2011). Interestingly, the massive amplification of RAE180 and other tandem repeats within male sex chromosomes occurred only in European species with multiple sex chromosomes, but not in the XX/XY1Y2 *R. hastatulus*.

*R. hastatulus* is a dioecious North American species possessing two chromosomal races: Texas (T) race (2n = 10), characterized by simple XX/XY sex chromosome system and North Carolina (NC) race with XX (2n = 8) in females and XY1Y2 (2n = 9) in males (Smith 1963). According to molecular studies by Navajas-Pérez et al. (2005a), the polymorphic sex chromosome system in this species is much younger than in *R. acetosa* (600,000 years vs. 12–13 mya). The co-occurrence of two different chromosome systems within a single species and relatively young age of *R. hastatulus* neo-sex chromosomes provides an ideal opportunity to study an early evolution of XX/XY1Y2 sex chromosome system in plants. To date, cytogenetic analysis of *R. hastatulus* has been limited, and the latest studies on the karyotype of this species were performed over 40 years ago (Smith 1964, Bartkowiak 1971).

The origin of polymorphic XX/XY1Y2 system in *Rumex* is still a controversial issue. There are two competing hypotheses: an autosome-heterosome translocation (Smith 1964) and a misdivision of an ancestral Y chromosome (Ruiz Rejon et al. 1994). Neither phylogenetic nor molecular data provide support to discriminate between these hypotheses, particularly in *R. acetosa* group, where the XX/XY plants are not available and similar families of repetitive sequences, originated from the same ancestral satellite-DNA, cover both the Y1 and Y2 chromosomes (Vyskot and Hobza 2004, Navajas-Pérez et al. 2005b). In *R. hastatulus*, where the evident reduction of chromosome number in the NC race was found, the autosome-sex chromosome translocation seems to be more probable. On the basis of the measurements of conventionally stained chromosomes, Smith (1964) suggested two successive translocations between the original XY chromosome pair and a pair of small autosomes. The postulated rearrangements, however, were not sufficiently documented, because the involved chromosome arms could not be distinguished using solid staining techniques.

The present study aimed at characterizing the two chromosomal races of *R. hastatulus*, and this goal was achieved using C-banding/DAPI and fluorescence in situ hybridization (FISH) techniques. We demonstrate that Y chromosomes of this species are enriched with DAPI-positive sequences, and that the reduction of chromosome number in NC race was accompanied by the switch in the localization of 5S rDNA from autosomes to heterosomes. Our results contradict earlier reports on the lack of heterochromatin within Y chromosomes of this species, allow for the unambiguous identification of autosomes involved in the autosome-heterosome translocation and provide useful chromosome landmarks for further studies on the karyotype and sex chromosome differentiation in this interesting species.

## Material and methods

### Plant material and chromosome preparation

Male and female *R. hastatulus* plants were grown from seeds in a greenhouse. Seeds of the North Carolina race collected from its natural population in the United States were kindly provided by Professor Spencer Barrett (University of Toronto, Canada). Seeds of the Texas race were obtained from The Royal Botanic Gardens, Kew, UK.

The radicles were collected, pretreated with saturated solution of α-bromonaphthalene for 24 h and fixed in a mixture of glacial acetic acid and absolute ethanol (1:3, v/v). Before squashing in 45 % acetic acid, fixed root tips were hydrolyzed in 1 M HCL at 60 °C for 13 min (for conventional chromosome staining with 0.1 % aqueous solution of toluidine blue) or macerated enzymatically (1 % pectinase + 1 % cellulase in citric buffer, ph 4.6) at 37 °C for 30 min (for differential chromosome staining with C-banding/DAPI method). C-banding/DAPI on squashed *R. hastatulus* preparations were conducted according to the procedure described by Grabowska-Joachimiak et al. (2011).

Chromosome observations were made using a Nikon Eclipse E800 microscope and the images were captured and processed with a Nikon DS-2MBWc camera and the NIS Elements software.

### DNA probes and fluorescence in situ hybridization

Two kinds of probes were used: (i) the 5S rDNA probe was generated by PCR amplification of a 410-bp *Bam*HI sub-clone of the 5S rDNA from the wheat clone pTa794 (Gerlach and Dyer 1980) and labelled also by PCR with tetramethyl-rhodamine-5-dUTP (Roche) by using universal M13 ‘forward’ (5′-CAG GGT TTT CCC AGT CAC GA-3′) and ‘reverse’ (5′-CGG ATA ACA ATT TCA CAC AGG A-3′) sequencing primers. The thermal cycling program was as follows: 94 °C for 1 min, 39 cycles of 94 °C for 40 s, 55 °C for 40 s, and 72 °C for 90 s and finally 72 °C for 5 min; (ii) the 26S rDNA probe, which was used for detection of 35S rDNA loci, was made by nick translation of a 2.3-kb *Cla*I sub-clone of the 26S rDNA coding region of *Arabidopsis thaliana* (Unfried and Gruendler 1990) with digoxigenin-11-dUTP (Roche). The conditions for this reaction were as follows: 15 °C for 95 min and 65 °C for 10 min.

The pre-treatment and denaturation of chromosome slides subjected to FISH experiments were carried out as follows: RNase treatment (37 °C for 1 h), 0.1× SSC at room temperature for 1 min, incubation of slides in 2× SSC at 65 °C for 30 min, 0.1× SSC at room temperature for 1 min, denaturation of slides in 0.07 N NaOH at room temperature for 1 min, 0.1× SSC at 4 °C for 1 min, 2× SSC at 4 °C for 1 min, and then the slides were dehydrated in an ethanol series at room temperature (Fritz et al. 1998, Rieder et al. 1998, with minor modifications). The FISH procedure was performed as described in detail by Książczyk et al. (2010). FISH images were acquired using an F-View II CCD camera attached to an Olympus BX 60 epifluorescence microscope. Image processing and superimpositions were done using Olympus Cell-F imaging software and Micrographx Picture Publisher software.

### DNA measurements

DNA content in young leaves of *Rumex* plants was estimated by flow cytometry. Nuclear samples were prepared as previously described (Błocka-Wandas et al. 2007). *Zea mays* line CE-777 (2C = 5.43 pg/nucleus Lysak and Doležel 1998) was used as internal standard. For each sample, (3000–5000) nuclei were analysed directly after preparation using a CyFlow SL Green (Partec GmbH, Münster, Germany) flow cytometer, equipped with a high-grade solid-state laser with green light emission at 532 nm, long-pass filter RG 590 E, DM 560 A, as well as with side (SSC) and forward (FSC) scatters. At least 10 plants from each cultivar were analysed. Histograms were evaluated using FloMax software (Partec GmbH, Münster, Germany). Nuclear DNA content was calculated using the linear relationship between the ratios of the *Rumex*/*Zea* peak positions on the histogram of fluorescence intensities.

## Results

**Fig. 1:**
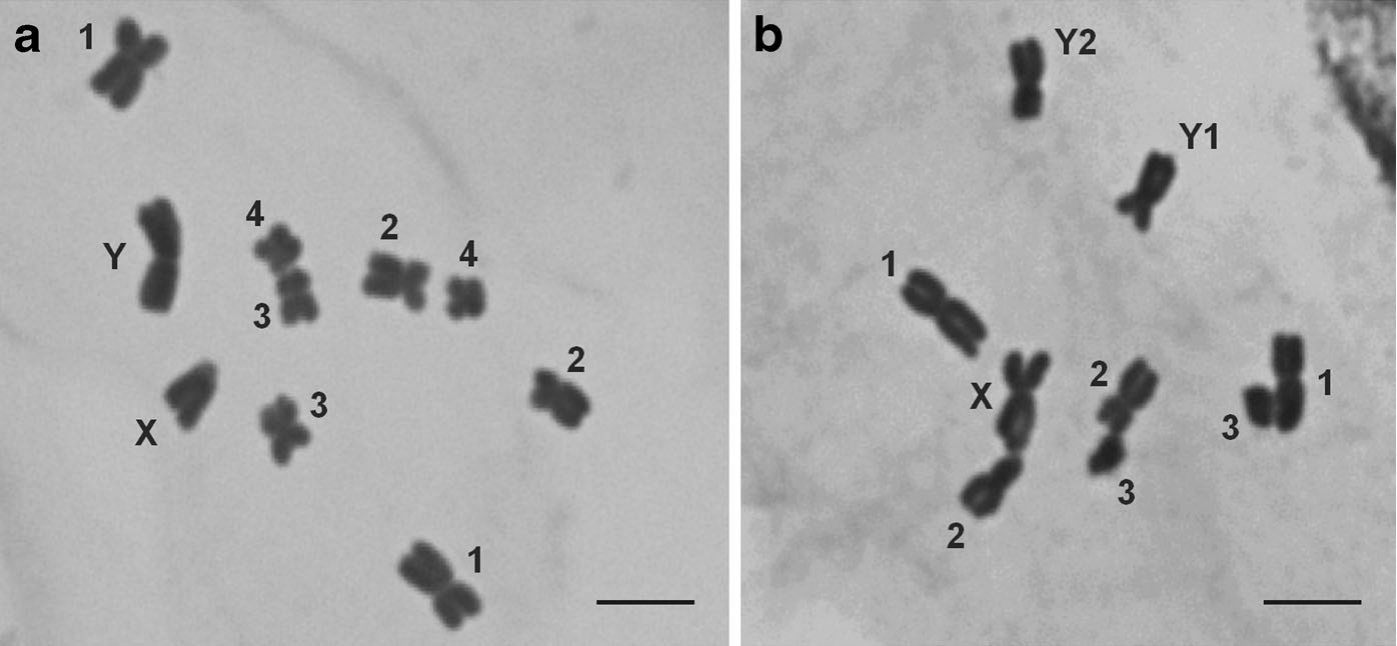
The morphology of toluidine blue-stained chromosomes of two *R. hastatulus* races. **a** Male metaphase plate of Texas race (2n = 10, XY). **b** Male metaphase plate of North Carolina race (2n = 9, XY1Y2). *Scale bar* = 5 μm

**Fig. 2:**
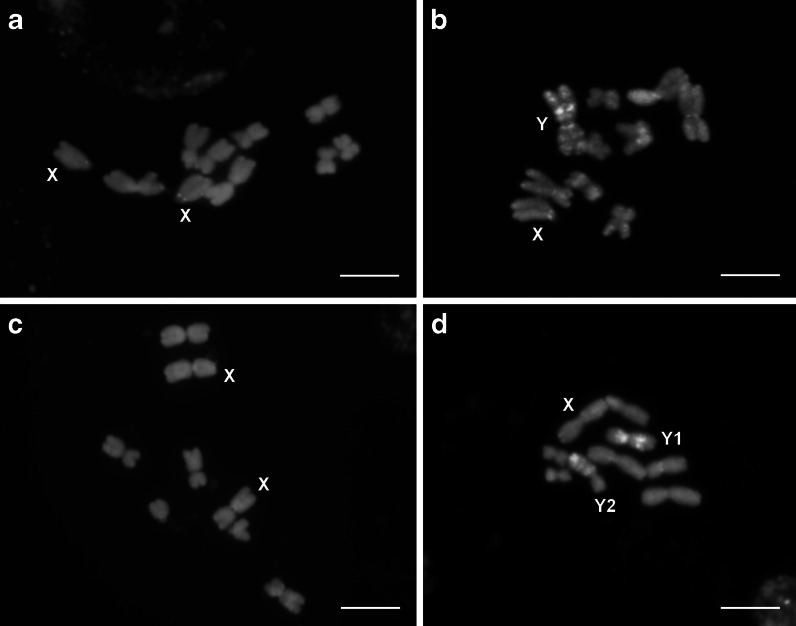
C-banding/DAPI-stained metaphase plates of *R. hastatulus*. **a**-**b** Texas race: female (**a**), male (**b**). **c**-**d** North Carolina race: female (**c**), male (**d**). *Scale bar* = 5 μm

**Fig. 3:**
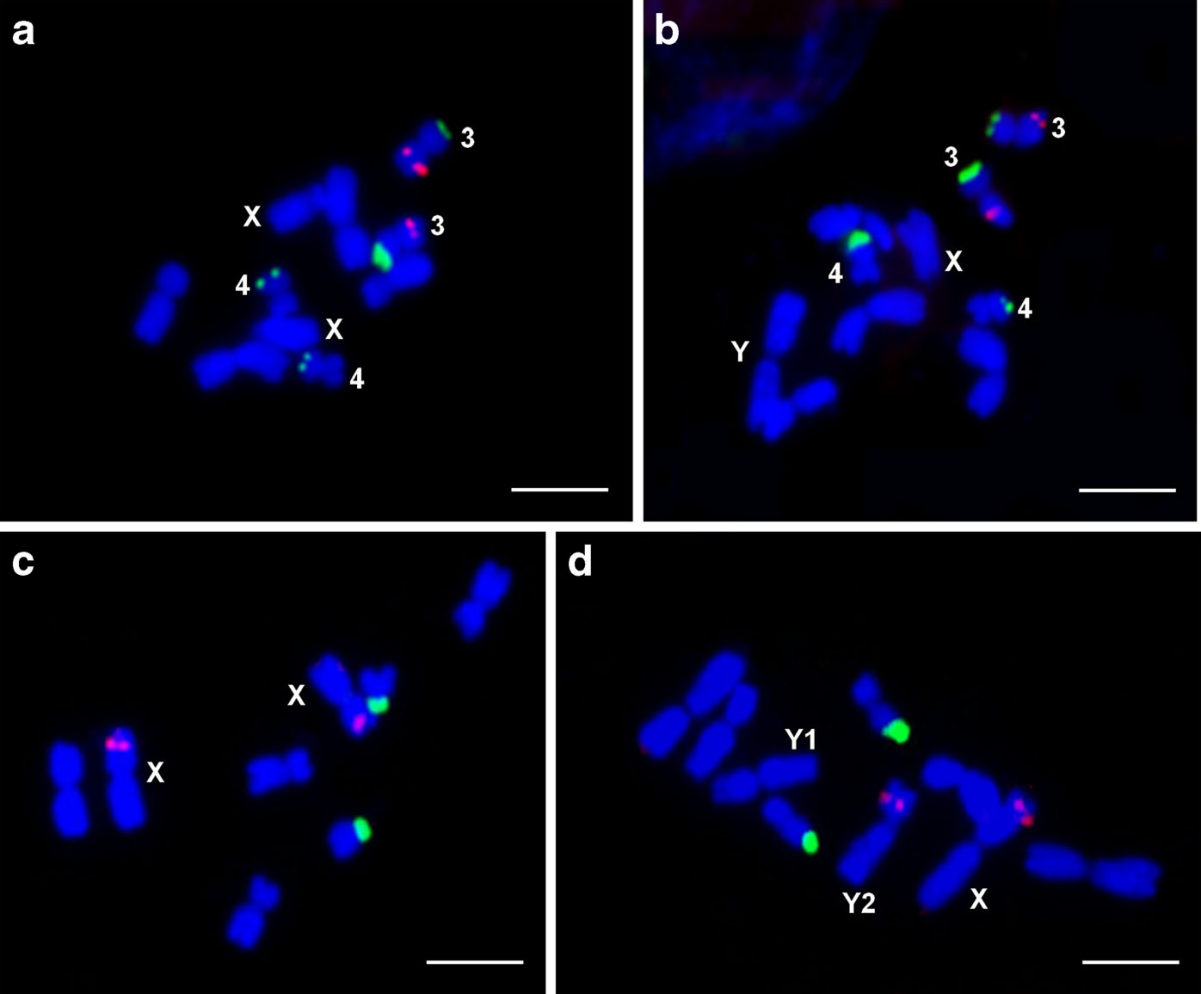
Double-FISH with rDNA probes on *R. hastatulus* chromosomes: 35S rDNA (green) and 5S rDNA (red). **a**-**b** Texas race: female (**a**), male (**b**), **c**-**d** North Carolina race: female (**c**), male (**d**). *Scale bar* = 5 μm

The autosome complement of the Texas race consisted of 4 metacentric chromosome pairs: two larger ones, clearly differing in length and centromere position and two smaller ones of similar morphology. The females of this race had two medium-sized X chromosomes, and the males had a heteromorphic pair of sex chromosomes. The Y chromosome was homobrachial and the largest in the complement, the X chromosome was extremely heterobrachial (Table 1). The sex chromosomes were well-distinguishable from each other and from autosomes in the conventionally stained preparations (Fig. 1a). After C-banding/DAPI (Fig. 2a, b), Y chromosome showed several interstitially located heterochromatic bands on both arms (Fig. 2b). When 35S and 5S rDNA were used as FISH probes, six hybridization sites were observed on the two small autosome pairs (Fig. 3a, b). Chromosome pair 3 showed 35S rDNA on the shorter arm and 5S rDNA on the longer arm, while chromosome pair 4 showed 35S rDNA on the shorter arm. The 35S rDNA signals were located terminally, whereas 5S rDNA signals interstitially. Considering the length differences and chromosome landmarks described here, all the chromosomes of T race of *R. hastatulus* were well-distinguishable from each other.

**Table 1:** Chromosome morphology in two *R. hastatulus* races

	Texas			N. Carolina	
	TL (μm, ±SD)	AR (±SD)		TL (μm, ±SD)	AR (±SD)
1	5.02 ± 0.71	1.22 ± 0.21	1	4.98 ± 0.73	1.17 ± 0.17
2	3.80 ± 0.44	1.73 ± 0.28	2	3.67 ± 0.43	1.64 ± 0.22
3	2.81 ± 0.24	1.25 ± 0.19			
4	2.35 ± 0.35	1.24 ± 0.13	3	2.20 ± 0.32	1.43 ± 0.38
X	3.40 ± 0.46	5.53 ± 0.79	X	5.36 ± 0.63	1.29 ± 0.23
Y	5.89 ± 0.61	1.21 ± 0.18	Y1	4.22 ± 0.59	1.24 ± 0.17
			Y2	3.77 ± 0.39	1.46 ± 0.28

For each race, chromosomes from 20 conventionally stained metaphase plates were measured

*TL* total chromosome length, *AR* arm ratio (L/S), *SD* standard deviation

**Fig. 4:**
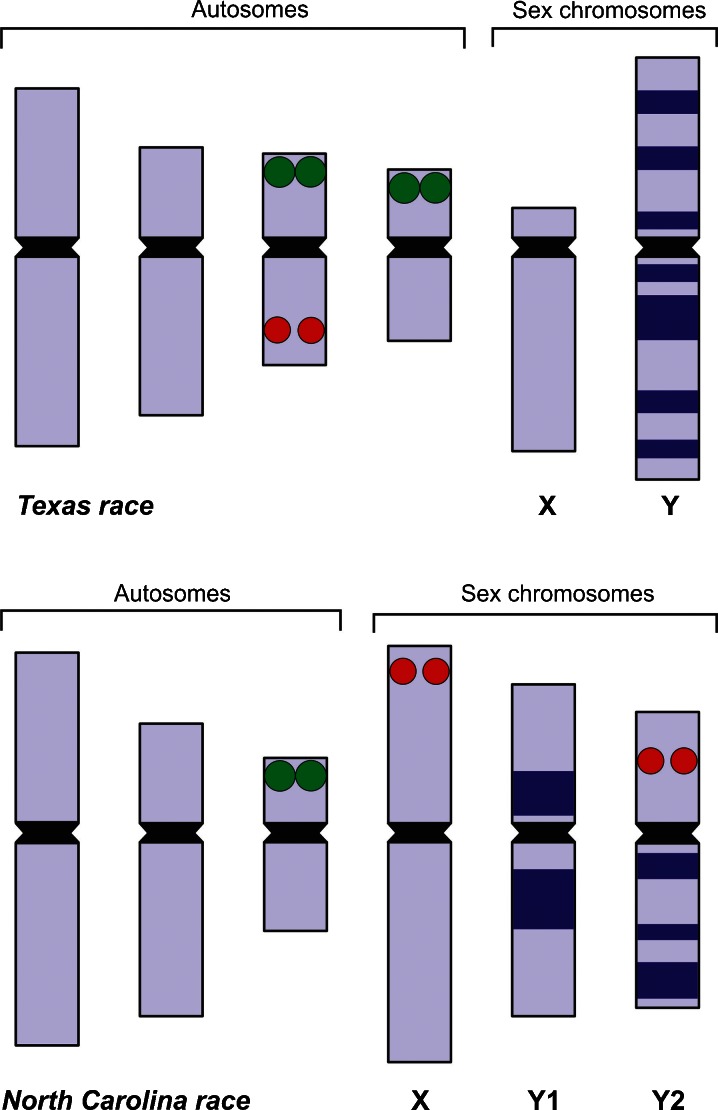
Ideograms of two *R. hastatulus* chromosomal races including C-banding/DAPI-positive bands on the Y chromosomes (*blue*), 35S rDNA sites (*green dots*) and 5S rDNA sites (*red dots*)

The autosome complement of the North Carolina race consisted of only three metacentric chromosome pairs, clearly differing in size. The females of this race had two large homobrachial X chromosomes. Male plants had three sex chromosomes: the X chromosome and two medium-sized Y chromosomes (Table 1). The Y chromosomes differed from one another in size and centromere position, but were hardly distinguishable from the larger autosomes in conventionally stained preparations (Fig. 1b). After C-banding/DAPI (Fig. 2c, d), Y chromosomes showed clearly visible heterochromatic bands, what differentiated them from all the other chromosomes in the complement. The Y2 chromosome possessed such bands only on the longer arm, the Y1 showed DAPI-positive segments on both arms (Fig. 2d). Double FISH revealed two large 35S and two tiny 5S rDNA sites in the metaphase plates of NC race. 35S rDNA signals were located on the smallest autosome pair, and the 5S rDNA sites were observed within shorter arms of two sex chromosomes, both in male and female metaphase plates. In females, two 5S rDNA signals were observed on both X chromosomes; in males, one signal was observed on X, whereas the other on the Y2 chromosome (Fig. 3c, d). Thanks to the different patterns of DAPI-positive segments and 5S rDNA signals, the sex chromosomes and their arms were easy to identify. Compared to the T race, the chromosome complement of NC race showed, apart from the loss of two autosomes (identified as the 3rd autosome pair of the Texas race), incorporation of 5S rDNA repeats to the sex chromosomes and loss of two 35S rDNA sites (Fig. 4).

Chromosome and nuclear DNA measurements in the two *R. hastatulus* races were summarized in Tables 1 and 2. The X chromosome size was 19.56 % of female genome (A + X) in T race and 33.07 % in NC race. The share of Y chromosome(s) in male genome (A + Y) of T race was 29.64 % and 42.41 % in male genome (A + Y1Y2) of NC race. The relative difference in 2C DNA content between male and female nuclei proved to be almost identical in both races (9.8 % and 9.7 %). On the other hand, the two races differed in nuclear DNA amount in males by 3.5 % and in females by 3.4 % (Table 2). It suggests loss of only a small amount of chromatin and preservation of size difference between male and female sex chromosomes in the derived karyotype.

**Table 2:** Comparison of genome/karyotype length (μm) and nuclear DNA amount (pg) in two races of *R. hastatulus*

	X^F^	X^M^	K^F^	K^M^	2C^F^	2C^M^
Texas	17.38	19.87	34.76	37.25	3.632	3.989
N. Carolina	16.21	18.84	32.42	35.05	3.511	3.852
Difference	1.17 (7.22 %)	1.03 (5.46 %)	2.34 (7.22 %)	2.20 (6.28 %)	0.121 (3.44 %)	0.137 (3.55 %)

*X* ^*F*^ female genome (A + X), *X* ^*M*^ male genome (A + Y/A + Y1Y2), *K* ^*F*^ female karyotype (2A + XX), *K* ^*M*^ male karyotype (2A + XY/2A + XY1Y2), *2C* ^*F*^ female DNA amount, *2C* ^*M*^ male DNA amount

## Discussion

The combination of C-banding/DAPI and FISH along with the conventional karyotype analysis provided deeper insight into the karyotype structure and more precise characterization of sex chromosomes in the two *R. hastatulus* races. The X and Y chromosomes in Texas race differed not only in size and morphology but also in the amount of heterochromatin. The occurrence of clearly visible DAPI-positive segments in Y chromosomes was not reported in any *Rumex* species with simple (probably ancestral) sex chromosome system (Cuñado et al. 2007, Navajas-Pérez et al. 2006, Navajas-Pérez 2012). Also in other well-studied XX/XY plant species, e.g. *Silene latifolia*, *S. dioica* and *Humulus lupulus*, Y chromosomes seem to be euchromatic (Grabowska-Joachimiak and Joachimiak 2002, Karlov et al. 2003). Among plants possessing well-differentiated (heteromorphic) XX/XY sex chromosomes, the only exceptions were heterochromatinised Y chromosomes of *Coccinia grandis* and *Cannabis sativa* (Sousa et al. 2013, Divashuk et al. 2014). Accumulation of heterochromatin within the male-specific region of the Y chromosome (MSY) was also revealed in *Carica papaya*, a species with homomorphic sex chromosomes (Zhang et al. 2008).

The Y chromosomes of North Carolina race were also marked by DAPI-positive bands. This is in contradiction to the statement by Quesada del Bosque et al. (2011) and Navajas-Pérez (2012) that the male sex chromosomes of this race lacked any contrastable DAPI+ bands and were still euchromatic. Most probably, heterochromatin revealed in the Y chromosomes of NC race was largely inherited from the Texas race, possessing an ancestral XX/XY sex chromosome system and heterochromatinised Y chromosome. A similar pattern of sex chromosome divergence in *R. hastatulus* was detected by Hough et al. (2014) on the molecular level. The authors showed that the older Y-linked genes that are shared between the XX/XY (T race) and XX/XY1Y2 (NC race) systems show clear signs of degeneration, in contrast to the Y-linked genes unique to the relatively younger XX/XY1Y2 system. The euchromatic character of the shorter arm of Y2 chromosome, representing the translocated autosome fragment confirms this assumption.

The observed interspecific differences in the heterochromatinisation of plant Y chromosomes may be a reflection of their different ages. However, the influence of other factors cannot be excluded, especially in some species with derived XX/XY1Y2 sex chromosome system, having close XX/XY relatives without heterochromatinised Y chromosomes. It cannot be ruled out that the extensive accumulation of repetitive sequences in Y chromosomes of *R. acetosa* and in *H. japonicus* has been accelerated by epigenetically regulated condensation of these chromosomes in interphase nuclei (Mosiołek et al. 2005, Grabowska-Joachimiak et al. 2011). It is interesting to date that all known plants possessing an XX/XY1Y2 sex chromosome system show accumulation of AT-rich sequences within their Y chromosomes (Ruiz Rejon et al. 1994, Shibata et al. 1999, Navajas-Pérez et al. 2006, 2009a, Cuñado et al. 2007, Mariotti et al. 2009, Navajas-Pérez 2012, Grabowska-Joachimiak et al. 2011, 2012).

Our FISH analyses demonstrated significant differences in the number and position of rDNA loci in the karyotypes of two *R. hastatulus* races. T race showed four 35S rDNA and two 5S rDNA clusters located autosomally, whereas NC race showed two 35S rDNA clusters on autosomes and two 5S rDNA sites on sex chromosomes: in females they were found on X chromosomes, in males on the X and Y2 chromosomes. In the absence of corresponding autosomal loci in the NC karyotype, such distribution ensures a balance in the amount of 5S rDNA in both sexes. A very similar phenomenon was observed in the catfish, *Harttia punctata*, possessing a polymorphic sex chromosome system X1X1X2X2/X1X2Y, where the only two 5S rDNA sites in the karyotype were observed on the X1 and Y chromosomes (Blanco et al. 2014).

The occurrence of ribosomal DNA (mainly associated with nucleolar organizer region) in sex chromosomes has been observed in several groups of organisms (da Cruz et al. 2011). In plants it is a rare phenomenon, observed so far exclusively in taxa with simple sex chromosome system. It was reported only in three angiosperm species, *Asparagus officinalis* (Deng et al. 2012), *Spinacia oleracea* (Lan et al. 2006) and *Carica papaya* (Zhang et al. 2010), all possessing primitive (homomorphic) sex chromosomes, and in the liverwort *Marchantia polymorpha* (Nakayama et al. 2001). All of these species, however, have analogous rDNA loci within the autosomes. In *A. officinalis*, two incipient sex chromosomes showed 5S and 35S rDNA clusters on their shorter arms, inherited from the ancestral autosome pair. On the other hand, both *M. polymorpha* and *S. oleracea* showed heterogenous location of 45S rDNA on sex chromosomes; it was detected on the X, but not on the Y chromosome. It is likely to be a result of the deletion which occurred in the Y chromosome (Newton 1984, Lan et al. 2006). Most probably, as in *Asparagus*, the ancestral configuration for these species consisted of rDNA located on each sex chromosome. Interestingly, in *C. papaya*, rDNA was detected on the Y, but not on the X chromosome. The presence of two small 5S rDNA loci associated with heterochromatic knobs within the papaya MSY suggested that amplification of rDNA contributed to the accumulation of heterochromatin in this chromosome region (Zhang et al. 2010).

The uneven distribution of 45S rDNA between sex chromosomes was also observed in some animal species. In fish, *Liobagrus styani*, the rDNA region in the Y chromosome is smaller in size than that in the X chromosome; in rainbow trout, frog *Gastrotheca riobambae* and in some *Drosophila* species, rDNA locus was preserved only in the X chromosomes (Schmid et al. 1983, Iturra et al. 2001, Roy et al. 2005, Chen et al. 2008). Generally, when the supernumerary 45S rDNA cluster occurred on the heterosome, it was always observed on the X chromosome. The attrition of functional sequences from the non-recombining, genetically isolated Y chromosomes can be considered a major force leading to heteromorphy between sex chromosomes (Graves 1995, Charlesworth and Charlesworth 2000).

*R. hastatulus* proved to be the only angiosperm species with rDNA located on heteromorphic sex chromosomes. We demonstrated that 5S rDNA in North Carolina race sex chromosomes were not inherited from the incipient chromosome pair, but derived from an ordinary pair of autosomes. The movement of rDNA from autosomal to heterosomal position has never been demonstrated in flowering plants, although the changes in the number and chromosomal position of rDNA clusters have already been reported between closely related species, and even within a given species (Schubert and Wobus 1985, Dubcovsky and Dvorak 1995, Pedrosa-Harand et al. 2006).

Our results confirm Smith’s (1964) supposition that the karyotype of North Carolina race might have originated from the karyotype of Texas race in the course of translocation between the small autosomes and sex chromosomes. We postulate that in the rearrangements of autosome sex chromosomes, the 3rd pair of autosomes of Texas race was involved, instead of the 4th pair of chromosome, what was supposed by Smith (1964). Most probably, the longer arms of autosomes containing 5S rDNA were translocated to sex chromosomes; the shorter arms or their major parts were eliminated. This is justified by lack of chromosomes of the Texas 3rd chromosome pair, reduction in the number of 35S rDNA loci as well as a slight reduction in size of the karyotype’s length in North Carolina race, roughly corresponding to the length of shorter arms of the 3rd autosome pair of the Texas race (2 × 1.25 μm = 2.5 μm). Smith (1964) considered the loss of the autosome fragment in his model as a difficult and unsolved problem and suggested that—for functional reasons—it must have been proceeded by ‘transfer of all essential autosomal material to the X-chromosome’. In our opinion, the loss of chromosome portions composed primarily of 35S rDNA should not be of functional significance because it was compensated by the presence of corresponding sequences located on the 4th pair of autosomes. Moreover, it is not known whether all the 35S rDNA loci are transcriptionally active in Texas race. In *R. acetosa*, 35S rDNA also occurs on two pairs of autosomes, but it has been demonstrated that in one of them it is silenced (Lengerova and Vyskot 2001).

C-banding/DAPI staining suggests that the breaking of the original Texas Y chromosome took place, and the part of autosome 3 was translocated to one of thus formed Y fragments. In this way, chromosome Y2 originated—with the longer C/DAPI-banded arm and the shorter euchromatic arm containing 5S rDNA. The analogous autosome fragment was translocated to the X chromosome. Chromosome Y1 of North Carolina race, possessing two large segments of DAPI-positive chromatin, originated from the second, most probably centric fragment of the original Y chromosome. Furthermore, breaking and inversion must have occurred because this chromosome is homobrachial now. The sequence of the described events is difficult to determine. It seems possible that the X chromosome received the autosomal addition above its pseudoautosomal region (PAR); and in the next meiosis, this autosomal fragment was able to cross over to the Y. Theoretically, it cannot be also excluded that evolution in *R. hastatulus* went in the opposite direction, i.e. the XX/XY1Y2 system occurring in North Carolina race was primeval. However, in *R. acetosa* (subg. Acetosa) and in *R. acetosella* (subg. Acetosella), 5S rDNA occurs only in autosomes; what might suggest that such localisation is ancestral for all dioecious *Rumex* species (Kim et al. 2006).

Our analyses did not allow us to establish which arm of the primary Y chromosome was developed to chromosome Y1 and which one to Y2. The distribution of DAPI-positive bands in male sex chromosomes in both *R. hastatulus* races do not correspond to each other (Fig. 4, see also Supplementary material) which might result from secondary changes, such as inversions or progressive heterochromatinisation of chromosomes Y1 and Y2. Furthermore, it is not known whether the primary centromere of the X chromosome was preserved, whether centric/acentric fragments of autosomes were translocated and what was the size of these fragments in both cases. Further research should allow for explanation of these issues.

Studies on polymorphic systems of sex chromosomes in plants showed that they might have derived from the XX/XY system on various ways. In *H. japonicus* (XX/XY1Y2), most probably, there was a translocation of the autosome to the primary X chromosome, and the original Y chromosome became Y1 and the other autosome gave rise to the Y2 (neo-Y) chromosome (Grabowska-Joachimiak et al. 2011). In *Silene diclinis*, reciprocal translocation between an autosome and the Y chromosome led to formation of a system with four sex chromosomes (X1, *X*2, Y1, and Y2) (Howell et al. 2009). Only in *R. hastatulus*, two autosome-heterosome translocations were evidenced, and the involved autosome pair was identified.

## Electronic supplementary material

Below is the link to the electronic supplementary material.

ESM 1 (JPEG 2691 kb)

## Abbreviations

DAPI: 4′,6-diamidino-2-phenylindole
FISH: Fluorescence in situ hybridization
NC: North Carolina
PAR: Pseudoautosomal region
PCR: Polymerase chain reaction
T: Texas
