# Supplementary figures and images for: Chromosome landmarks and autosome-sex chromosome translocations in Rumex hastatulus, a plant with XX/XY1Y2 sex chromosome system
Source: Chromosome Res. 2014 Nov 14;23(2):187–97. doi: 10.1007/s10577-014-9446-4 (PMC4430600; doi:10.1007/s10577-014-9446-4)

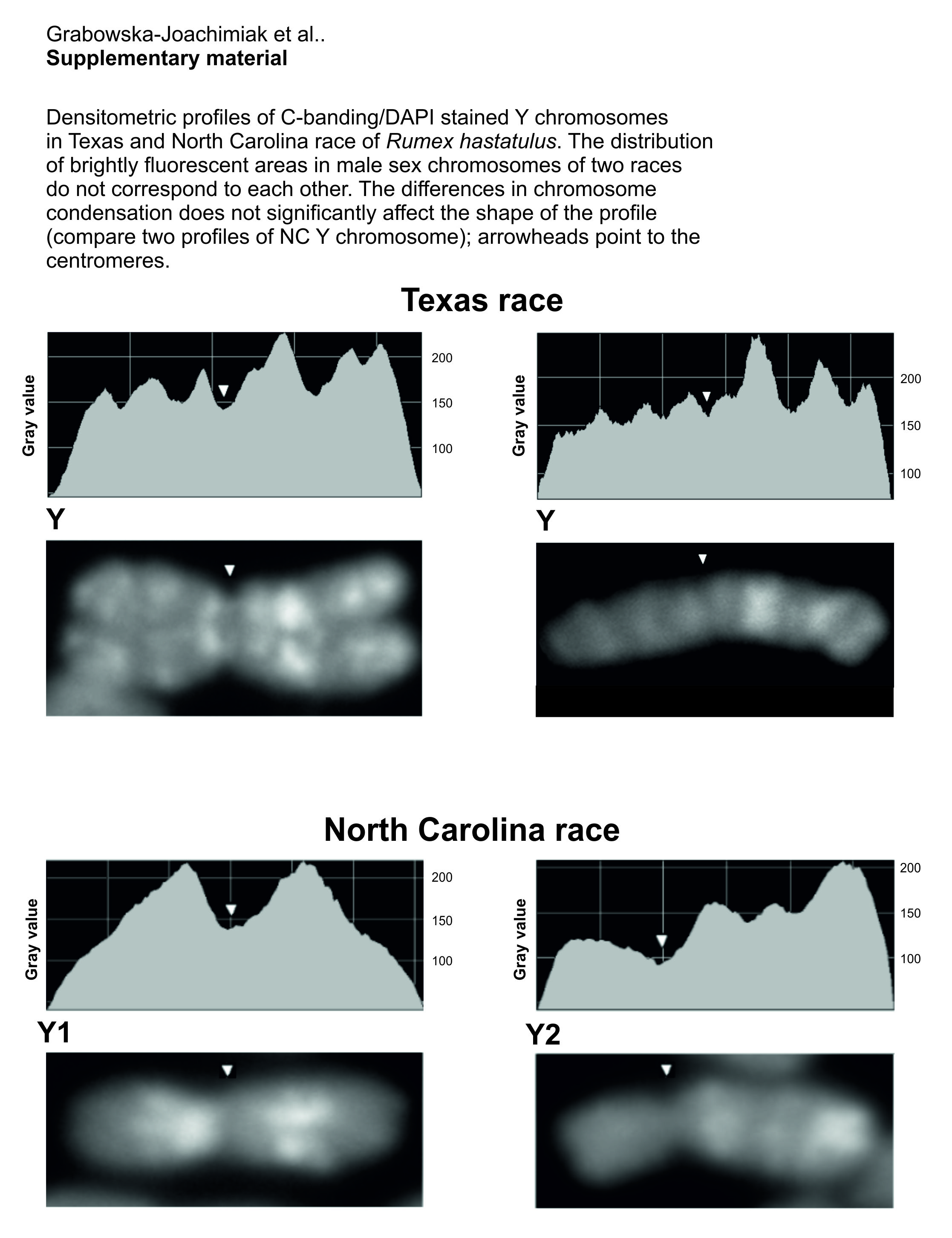

Supplement: Supplementary file 1 — (JPEG 2691 kb) [file 10577_2014_9446_Fig5_ESM.jpg]
